# Carrier-dependent magnetic anisotropy of cobalt doped titanium dioxide

**DOI:** 10.1038/srep07496

**Published:** 2014-12-16

**Authors:** Bin Shao, Min Feng, Xu Zuo

**Affiliations:** 1 State Key Laboratory of Low-Dimensional Quantum Physics, and Department of Physics Tsinghua University, Beijing 100084, China; 2 School of Physics, Nankai University, Tianjin 300071, China; 3 College of Electronic Information and Optic Engineering, Nankai University, Tianjin 300071, China

## Abstract

Using first-principles calculations, we predict that the magnetic anisotropy energy of Co-doped TiO_2_ sensitively depends on carrier accumulation. This magnetoelectric phenomenon provides a potential route to a direct manipulation of the magnetization direction in diluted magnetic semiconductor by external electric-fields. We calculate the band structures and reveal the origin of the carrier-dependent magnetic anisotropy energy in *k*-space. It is shown that the carrier accumulation shifts the Fermi energy, and consequently, regulates the competing contributions to the magnetic anisotropy energy. The calculations provide an insight to understanding this magnetoelectric phenomenon, and a straightforward way to search prospective materials for electrically controllable spin direction of carriers.

Diluted magnetic semiconductor (DMS), exhibiting both ferromagnetism and semiconducting properties, has been considered a potential candidate of spintronics^1^. It provides a potential route to adding the spin degree of freedom to conventional charge-based electronic devices, for example, adding magnetic recording capability to current semiconductor information processing unit. However, there are two major challenges, one is how to raise the Curie temperature (*T*_C_), and the other is how to control the magnetization direction by using solely electric-field or voltage, which can be also called as magnetoelectric effect^2^. Traditionally, magnetoelectric effect can be observed in hexaferrites^3^,^4^, multiferroics^5^ and other materials. Noteworthy, for DMS, the electric-field-induced room-temperature ferromagnetism recently has been demonstrated in cobalt-doped titanium dioxide (Co:TiO_2_)^6^, where the ferromagnetic exchange interaction is mediated by carriers and controlled by electric-field. Thus, the remaining issue is how to manipulate the magnetization direction.

Conventionally, the magnetization direction is controlled by magnetic-field, which is unsuitable for ultrahigh-density magnetic storage and integrated electronic devices. Therefore, explorations aiming at manipulation of magnetization direction directly by electric-field have emerged. Early experiment^7^ revealed that the coercive force *H*_C_ depends on the carrier density controlled by electric-field in Mn-doped InAs. This phenomenon implies a correlation between magnetic anisotropy and carrier density in DMS. Further work^8^ exhibited the rotation of magnetization direction by applying electric-field in Mn-doped GaAs, showing the direct connection between magnetic anisotropy and carrier density.

When an external electric-field is applied or dopants atoms are added in DMS, there will be extra electrons or holes, yielding a shift of the Fermi energy (*E*_F_). Because magnetic anisotropy energy (MAE) is mainly determined by the band structure near the *E*_F_, the shift will impact the MAE. Early first-principles calculations of MAE in transition metal bulk and thin films have shown this effect by the so-called electron-filling technique, where electrons are added to or removed from the system under investigation^9^,^10^,^11^. We expect that carrier accumulation may also impact the MAE in DMS. Moreover, it will be easier to realize carrier accumulation and observe its impact on MAE in DMS materials.

In this work, our numerical calculations demonstrate that carrier accumulation can flip the sign of MAE in a typical DMS material, Co:TiO_2_. A straightforward but insightful analysis, based on the detailed band structure near *E_F_* instead of single-ion anisotropy theory^12^,^13^, is proposed to elucidate the carrier-dependent MAE.

## Results

**Figure 1:**
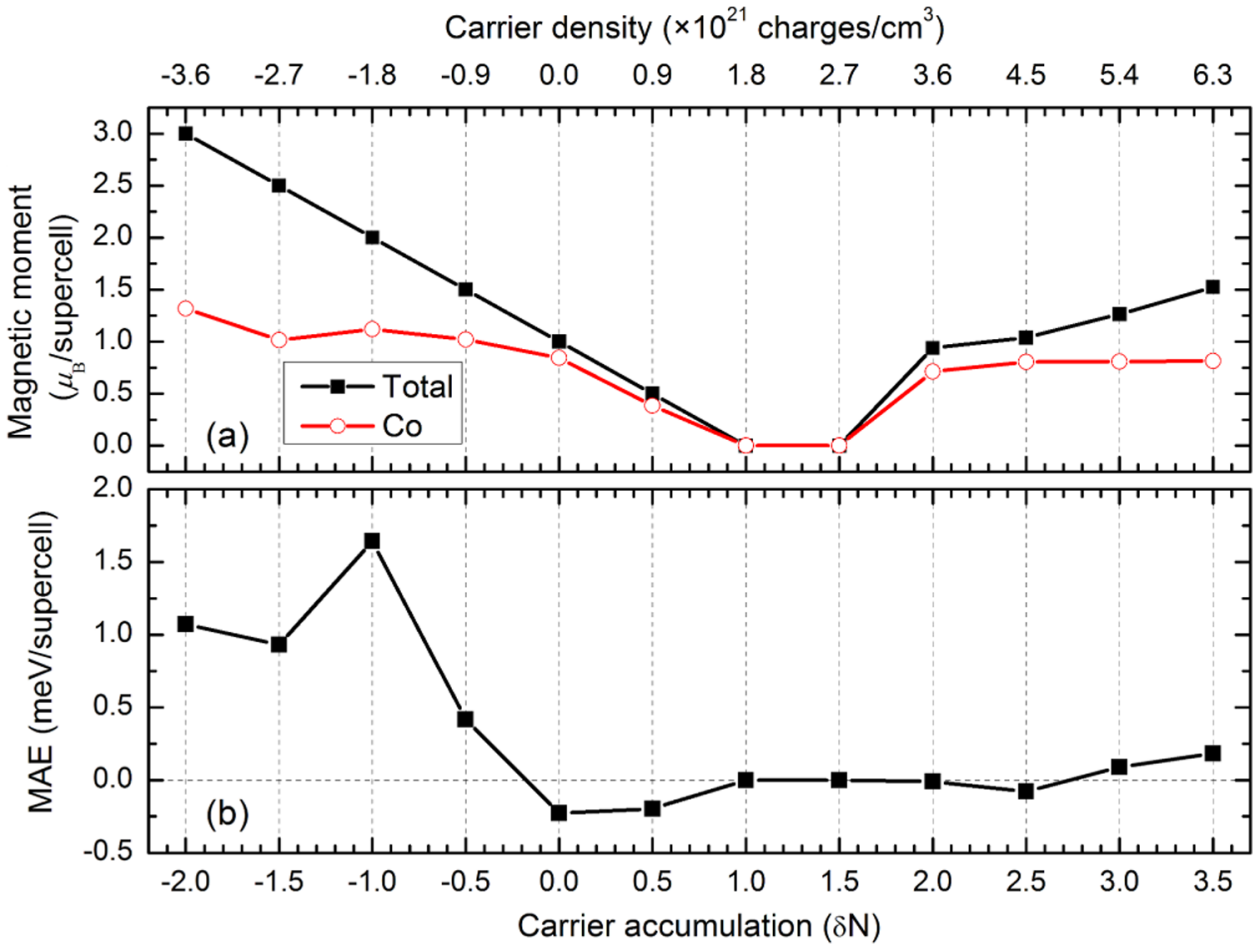
The impact of carrier accumulation on (a) the total magnetic moment, magnetic moment on Co atom and (b) MAE of Co_0.0625_Ti_0.9375_O_2_. |*δN*| = 1.0 corresponds to a carrier density of 1.8 × 10^21^ charges/cm^3^.

Fig. 1 shows the MAE and the magnetic moment of Co:TiO_2_ (Co_0.0625_Ti_0.9375_O_2_) as a function of the carrier accumulation (*δN*), where positive (negative) *δN* means that electrons are added (removed). The maximum carrier accumulation discussed in our work is *δN* = 3.5, which corresponds to 6.3 × 10^21^ electrons per cm^3^. It is obvious that both the MAE and the magnetic moment sensitively depend on carrier accumulation. In neutral Co:TiO_2_, the MAE is negative, implying that the anatase *ab*-plane is the easy plane of the magnetization. However, when electrons are removed, the easy-axis rotates out of the *ab*-plane, and the *c*-axis becomes the easy-axis. The magnitude of MAE increases and reaches maximum value at *δN* = −1.0, about 1.6 meV per supercell (4.65 × 10^6^ erg/cm^3^), which is comparable to that of hcp Co (5 × 10^6^ erg/cm^3^)^14^. Furthermore, our calculations show that the ferromagnetic state is more stable and that the Curie temperature (*T*_C_) from first-principles is 166°K at a concentration of 6.25% when *δN* = −1.0 (Supplement Information, Section I). It should be noted that the Co concentration can be as high as 10% without precipitation in experiment^6^. The calculated MAE consisting of several tens of Co ions in a tiny area (5 nm^2^) will be strong enough to resist the thermal fluctuation at room temperature (Supplement Information, Section II). In addition, when electrons are added, the magnitude of easy plane magnetic anisotropy diminishes and then vanishes for *δN* = 1.0 to 2.0. When more electrons are accumulated, the MAE is weak, less than 0.25 meV. According to our calculations, the magnetization direction will switch from planar easy axis to uniaxial axis by varying carrier density, which can be achieved by carrier accumulation and controlled by external electric-field.

When *δN* decreases from 0 to −2.0, the total magnetic moment of the supercell (*M*_tot_) linearly increases from 1.0 to 3.0 *μ*_B_, however, the local magnetic moment on Co (*M*_Co_) remains steady. In contrast, when *δN* varies from 0 to 1.0, both *M*_tot_ and *M*_Co_ decrease to 0 *μ*_B_, indicating the system becomes non-magnetic. The close coincidence of the two curves suggests the magnetic moment is mainly due to the Co ion at this range of *δN*. When *δN*, further, increases from 1.5 to 2.0, the system regains magnetic and M_tot_ drastically increases to 1.0 *μ*_B_. When more than 2 electrons are accumulating in a unit cell, the *M*_tot_ keeps increasing up to 1.5 *μ*_B_, while *M*_Co_ stays constant.

**Figure 2:**
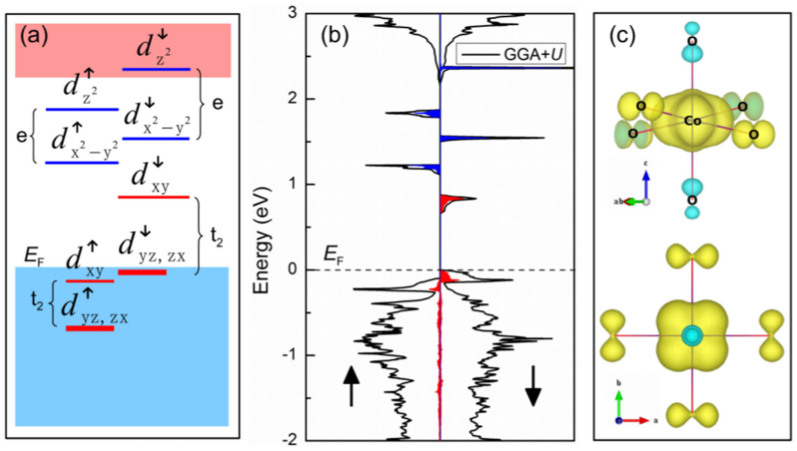
(a) Energy-level diagram, (b) density of states and (c) spin density map for neutral Co_0.0625_Ti_0.9375_O_2_ with GGA + *U* (*U*_Ti−*d*_ = 2 eV, *U*_Co−*d*_ = 2 eV) approach. In density of states, red filled plot and blue filled plot refer to Co-*d* *t*_2_ and Co-*d* *e*, respectively. The spin density map indicates that the magnetic moment is mainly contributed from the unoccupied *d*_xy_ orbital in minority spin.

To understand the impact of carrier accumulation on magnetic moment and MAE, we first check the electronic structure of Co:TiO_2_. The density of states [Fig. 2(b)] for neutral Co:TiO_2_ with GGA + *U* (generalized gradient approximation plus on-site Coulomb repulsion) shows that the band gap of pristine TiO_2_ is preserved, and that the impurity bands lie in the gap. The well isolated impurity bands are mainly from the Co *d*-orbitals, which hybrid with the O *p*-orbitals near the valence band maximum (VBM). The hybridization between the Co and O orbitals leads to slight magnetization of O atom as shown in the spin density map [Fig. 2(c)]. The majority of Co *t*_2_ manifold is completely occupied, while the minority of *t*_2_ manifold is split into two occupied doubly-degenerate states (*d*_xz,yz_) and an empty non-degenerate state (*d*_xy_). The insulator ground state is different from the half-metallic ground state predicted by GGA^15^,^16^,^17^. The total magnetic moment of the cell (*M*_tot_ = 1.0 *μ*_B_) and the spin density map [Fig. 2(c)] of Co atom in a shape of *d*_xy_ orbital suggest a low-spin state, 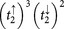, which is consistent with previous beyond density functional theory results^15^,^17^.

**Figure 3:**
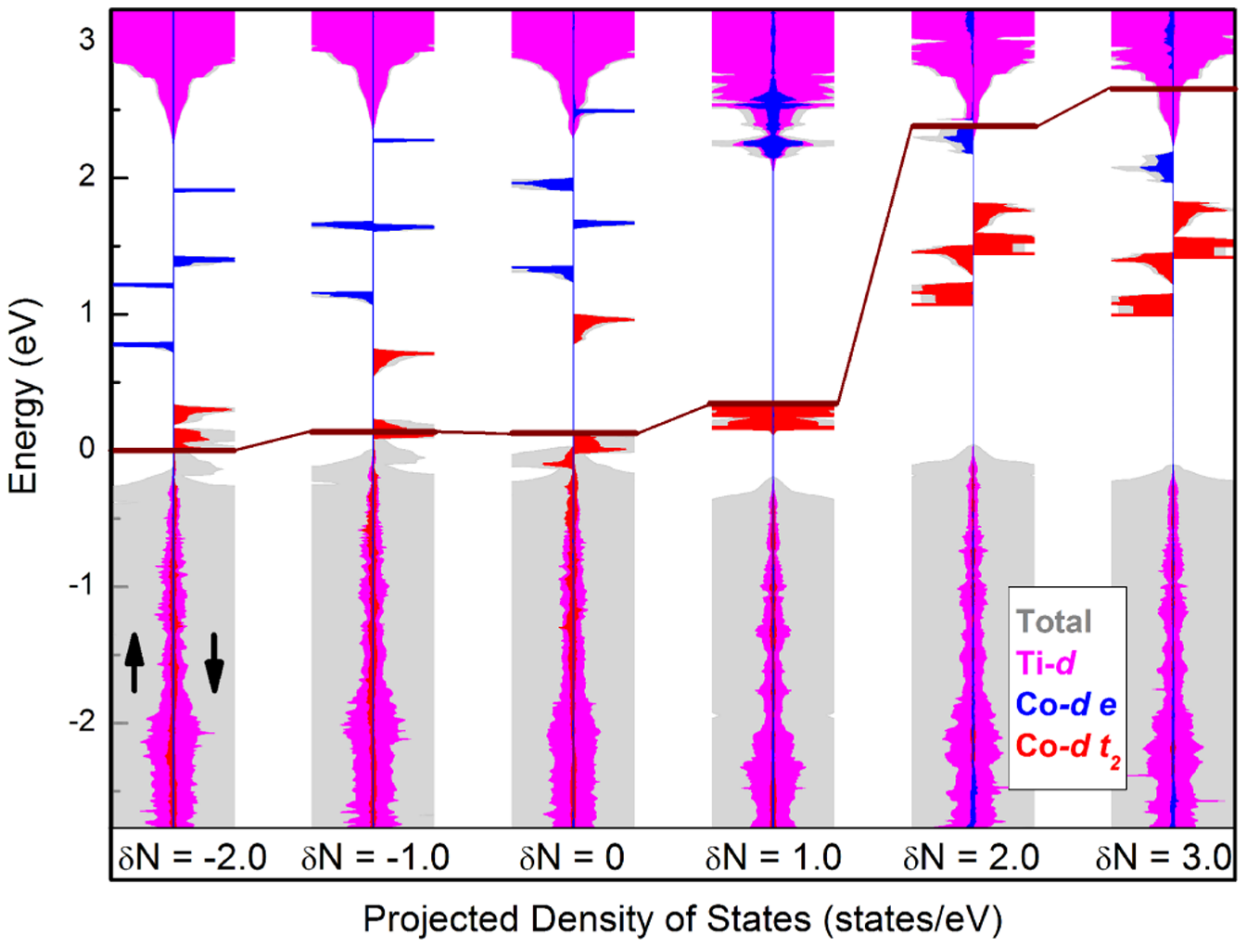
The projected density of states (PDOS) of Co_0.0625_Ti_0.9375_O_2_ with different *δN*. All DOS are aligned to the deep O-*s* states (~−17 eV). The carrier accumulation causes an obvious shift of the *E*_F_ (horizontal wine line). In PDOS, gray shaded plot, magenta filled plot, blue filled plot and red filled plot refer to total, Ti-*d*, Co-*d* *e*, and Co-*d* *t*_2_, respectively.

The carrier accumulation causes an obvious shift of the *E*_F_ (Fig. 3), if the projected density of states with different *δN* are aligned to the deep O-*s* state. When one electron is added (*δN* = 1.0), the non-degenerate state (*d*_xy_) in minority spin is occupied, and the system becomes non-magnetic. In addition, the Co-*d* *e* manifold is pushed into the conduction band (CB) of TiO_2_ host. When two electrons are added, the *E*_F_ shifts further into the host conduction band, and results in the so-called “band-filling effect”, i.e., the host conduction band minimum (CBM) will be first occupied and then the *e* manifold of Co will be partially filled^18^. Similarly, when electrons are removed, only part of them will be removed from the Co *t*_2_. Therefore, *M*_Co_ approximately maintains a constant value with *δN* < 0 and *δN* > 1.5 [Fig. 1(a)], which is called the negative-feedback charge regulation, Ref. 19.

It is well-known that the spin-orbit coupling (SOC) interaction of 3*d* transition metal elements is much weaker than the crystal-field split, and that MAE can be estimated by single-ion anisotropy theory^12^,^13^. However, the system becomes metallic when *δN* < 0 and *δN* > 1.0, and consequently, the charge state of Co is no longer well-defined. Then, the traditional single-ion anisotropy theory, where a well-defined charge state of magnetic ion is prerequisite, might not be suitable under this circumstance. More than that, when the Co concentration is high, the interaction between them will “bend” the Co-*d* levels, where the single-ion anisotropy theory is no longer applicable.

On the other hand, the MAE can be obtained by integrating the net contributions of the SOC interaction between the 3*d* sub-bands in *k*-space^10^,^20^,^21^. The contributions from degenerate and non-degenerate perturbations result in the first-order and second-order contributions, respectively. For the non-degenerate part, the contribution to MAE depends on the interaction between the occupied and empty states^10^. It should be noted that the degenerate contribution could be as important as the non-degenerate part, although the degeneracy may occur only in a small portion of the Brillouin zone^22^. Thus, we compare the unperturbed band structures near the *E*_F_ for different *δN*.

**Figure 4:**
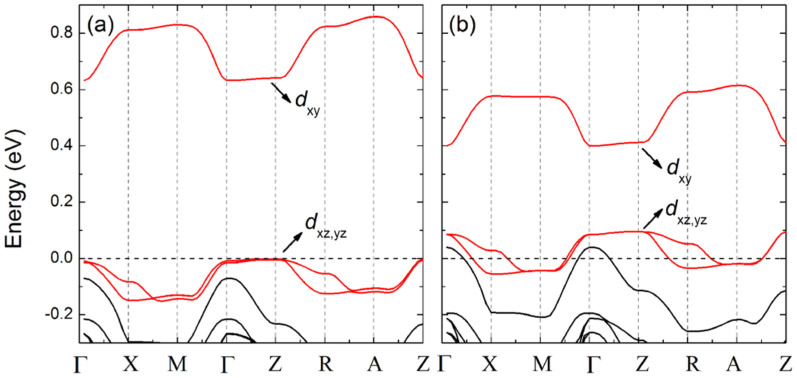
Band structure of minority spin near the *E*_F_ (horizontal dash line at 0 eV) along the edge of the irreducible Brillouin zone (IBZ) without SOC, (a) *δN* = 0 and (b) *δN* = −1.0. The Co-*d* bands are plotted in red.

Because of the fully occupied *t*_2_ manifold in majority spin and the low-spin configuration, the contribution to MAE is dominated by the spin-conservation terms of minority spin, and that from the spin-flip terms is negligible^10^,^21^,^23^. Therefore, we only plot band structures of minority spin near the *E*_F_ for *δN* = 0 and −1.0 in Fig. 4(a) and (b) without spin-orbit coupling, respectively. It is obvious that the *t*_2_ manifold splits into a non-degenerate state (*d*_xy_) and a doubly-degenerate state (*d*_xz_, *d*_yz_), because of the local D_2d_ symmetry of the Co dopant. The doubly-degenerate state further splits along some directions, e.g. Z to R, because of the dispersion, when the translation symmetry is considered. As a result, the contribution to MAE can be divided into three categories: (i) the SOC interaction between occupied *d*_xz_, *d*_yz_ and empty *d*_xy_, (ii) the SOC interaction between occupied *d*_xz_(*d*_yz_) and empty *d*_yz_(*d*_xz_), and (iii) the SOC interaction inside the doubly-degenerate state *d*_xz,yz_. The sign of MAE can be estimated by summing above three contributions.

For (i) the SOC interaction between the occupied *d*_xz,yz_ and empty *d*_xy_ that have different magnetic quantum numbers, the perturbation is through ***L_x_*** operator and yields a negative contribution (the easy axis is in the *ab*-plane)^11^. For (ii) the SOC interaction between occupied *d*_xz_(*d*_yz_) and empty *d*_yz_(*d*_xz_) that share the same magnetic quantum numbers, the perturbation is through ***L_z_*** operator and yields a positive contribution (the easy axis is the *c*-axis)^11^. Note that the energy split between *d*_xz_ and *d*_yz_ is almost a tenth of that between *d*_xz,yz_ and *d*_xy_. The contribution from (ii) can be 10 times larger than (i). For (iii) degenerate state, ***L*** is unquenched. In the subspace spanned by |*xz*〉 and |*yz*〉, the SOC Hamiltonian can be written as


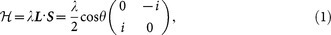


where *θ* is the angle between ***L*** and ***S*** and also the angle between spin axis and the *c*-axis, because ***L*** is always along the *c*-axis in the calculation. The eigenvalues of the Hamiltonian are


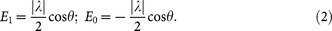


If there is only one electron in these states, e.g. *δN* = −1.0, the low-lying level *E*_0_ will be occupied. As a result, the energy of the system depends on *θ* with the minimum at *θ* = 0, i.e. the easy-axis is parallel to the *c*-axis-uniaxial. Therefore, we conclude that the contribution from the degenerate states to the MAE is positive.

According to above analysis, the results of MAE for *δN* = 0 and *δN* < 0 can be explained qualitatively. For *δN* = 0, there is only perturbation of category (i), resulting in the moderate negative MAE. For *δN* = −1.0, there are all three contributions, where both categories (ii) and (iii) are positive, and the magnitude of category (ii) is larger than (i). Thus, the MAE is positive.

**Figure 5:**
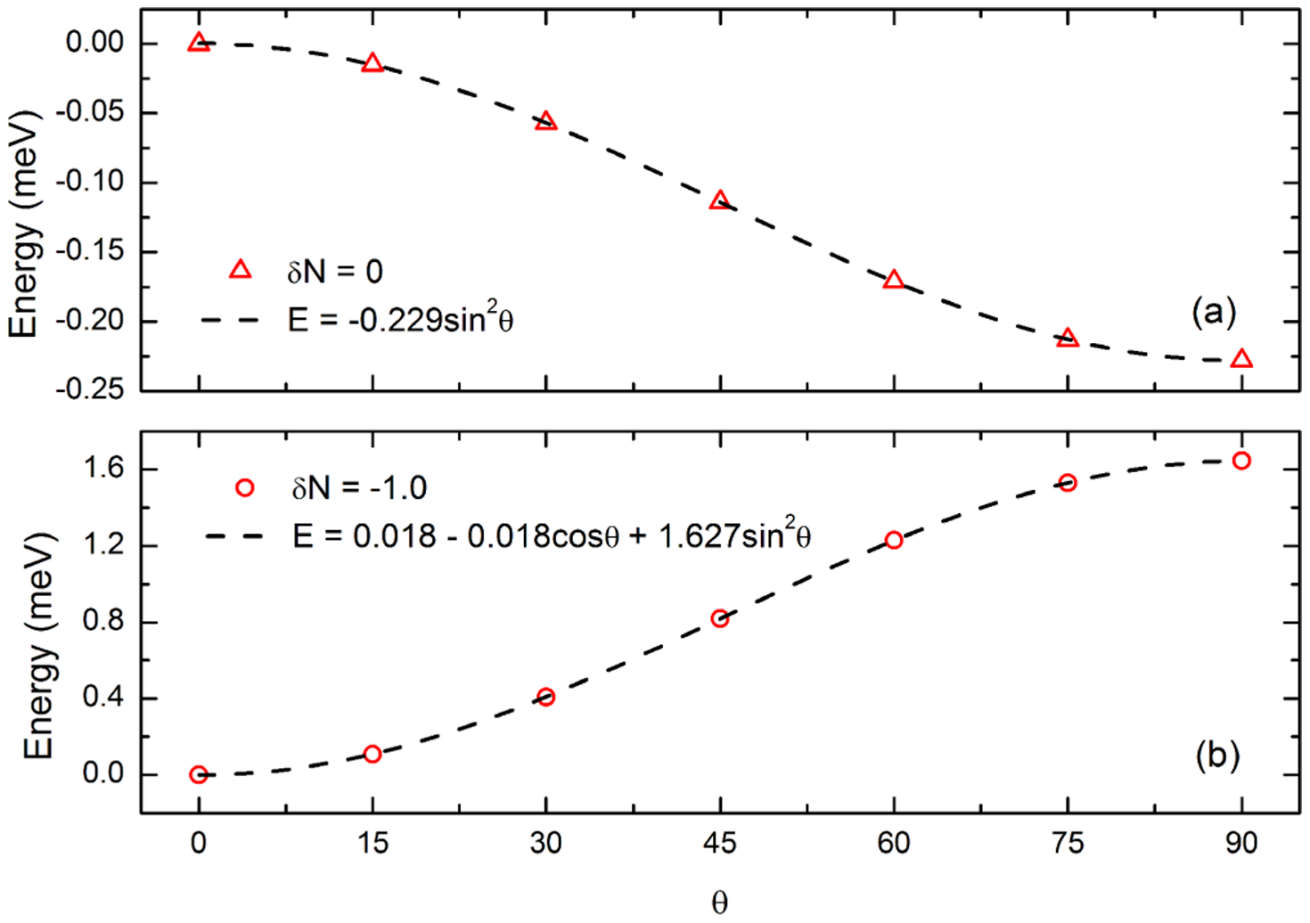
The dependence of the total energy on the angle (*θ*) between the quantization axis of spin and the anatase *c*-axis, (a) *δN* = 0 and (b) *δN* = −1.0.

The dependence of the total energy on the angle (*θ*) between the quantization axis of spin and the anatase *c*-axis (Fig. 5) supports our argument quantitatively. The coefficient of cos*θ* indicates the contribution from degenerate perturbation (first order perturbation). For *δN* = 0, it is less than 10^−6^ eV, implying the contribution from degenerate perturbation can be ignored. The sign of the coefficient of sin^2^ *θ* includes the competing contributions from the non-degenerate perturbation (second order perturbation).

For *δN* > 1.0, the MAE decreases by one order of magnitude. As shown in Fig. 3, when *δN* > 1.0, the *e* manifold becomes partially occupied. In fact, there will be a strong Jahn-Teller distortion for *d*^7^ electronic configuration in low-spin state in octahedral crystal field. Consequently, the MAE will be weak, because Jahn-Teller effect increases the split between occupied and empty states in general. For example, it increases the split to about 1 eV for *δN* = 2.

In fact, because of intrinsic defects, e.g. oxygen vacancy, Co:TiO_2_ usually exhibits a characteristic of n-type in experiment^24^,^25^,^26^,^27^. In this circumstance, it is difficult to enhance MAE by adding p-type carriers according to our predictions. However, it has been reported that by incresing the concentration of Cr dopants in TiO_2_, its electric conduction can be altered from n-type to p-type^28^,^29^,^30^. Therefore, by using of Cr and Co codoping, it might be possible to achieve the case of our calculations.

In summary, our first-principles calculations predict that the MAE in Co:TiO_2_ can be controlled by carrier accumulation. That magnetoelectric phenomenon in this typical DMS allows an efficient manipulation of the magnetization direction directly by external electric-field or voltage. To interpret the impact of carrier accumulation on the MAE, the electronic structures are calculated and examined. A self-regulated feedback effect of local magnetic moment on Co has been discovered. The MAE is discussed in *k*-space based on the band structure near the Fermi energy. By applying perturbation method, the contributions to the MAE have been divided into three categories in opposite signs and different magnitudes. The shift of the Fermi energy induced by carrier accumulation regulates the contributions from competing categories, and consequently determines the sign and magnitude of the MAE.

## Methods

By using the ionic liquid instead of the conventional solid insulator as the gate insulator, an extremely high carrier concentration, 4 × 10^21^ charges/cm^3^ equivalent to 2.5 electrons per La_0.8_Ca_0.2_MnO_3_ unit cell area, can be achieved in manganite^31^. Moreover, a carrier density of the order of ≈10^22^ cm^−3^ has been reported in nanogranular metallic Fe-oxygen defficient TiO_2−*δ*_ composite films^32^. Actually, besides applying an external electric field, the carrier concentration can be also tuned by adding dopants atoms in experiment^25^,^26^,^27^. However, in the latter case, dopants atoms might introduce extra defect levels and complicate the problem. Therefore, in our calculations, carrier accumulation was investigated upto a high but realistic density (~10^21^ charges/cm^3^) and simulated by modifying the total number of electrons per supercell, assuming a homogeneous background charge. The structure was optimized at each carrier density, before the calculation of MAE.

Based on density functional theory (DFT), first-principles calculations were carried out on Co-doped TiO_2_ anatase using Perdew-Burke-Ernzerhof (PBE) parameterization^33^ of generalized gradient approximation (GGA) as implemented in VASP package^34^. The primitive anatase cell was fully optimized. Then, a 2 × 2 × 1 supercell was created with one Ti atom substituted by Co (Co_0.0625_Ti_0.9375_O_2_), and all atomic positions were allowed to relax. After the optimization, GGA + *U* (GGA plus on-site Coulomb repulsion) approach^35^ was employed in the electronic structure calculations. We applied extra Coulomb repulsion to Ti-*d* orbital (2 eV) and Co-*d* orbital (2 eV)^36^. The *U* parameter on Ti-*d* has been carefully checked from 0 to 6.0 eV. The conclusion in this work are not sensitive to the choice of *U*_Ti−*d*_. The plane wave cut-off energy was 500 eV. The tetrahedron method with a 5 × 5 × 4 *k*-mesh grid was employed for the integration in Brillouin zone. The accuracy of electronic iterations was up to 10^−6^ eV. The MAE, merely considering the contribution from spin-orbit coupling, was calculated following the Force theorem^9^ as MAE = *E*_[100]_ − *E*_[001]_, where *E*_[100]_ and *E*_[001]_ were the total energies with magnetization directions along [100] and [001], respectively.

## Author Contributions

*Ab initio* calculation was performed by B.S., B.S. and M.F. conducted the post-analysis. B.S. and X.Z. wrote the main manuscript text and prepared all figures. All authors contributed to discussions and reviewed the manuscript.

## Supplementary Material

Supplementary Information Carrier-dependent magnetic anisotropy of cobalt doped titanium dioxide

## References

[b1] Sato K. *et al.* First-principles theory of dilute magnetic semiconductors. Rev. Mod. Phys. 82, 1633–1690 (2010).

[b2] Fiebig M. Revival of the magnetoelectric effect. J. Phys. D: Appl. Phys. 38, R123 (2005).

[b3] Vittoria C., Somu S. & Widom A. Tensor properties of the magnetoelectric coupling in hexaferrites. Phys. Rev. B 89, 134413 (2014).

[b4] Ebnabbasi K., Vittoria C. & Widom A. Converse magnetoelectric experiments on a room-temperature spirally ordered hexaferrite. Phys. Rev. B 86, 024430 (2012).

[b5] Eerenstein W., Mathur N. D. & Scott J. F. Multiferroic and magnetoelectric materials. Nature 442, 759–765 (2006). 16915279 10.1038/nature05023

[b6] Yamada Y. *et al.* Electrically induced ferromagnetism at room temperature in cobalt-doped titanium dioxide. Science 332, 1065–1067 (2011). 21617071 10.1126/science.1202152

[b7] Chiba D., Yamanouchi M., Matsukura F. & Ohno H. Electrical manipulation of magnetization reversal in a ferromagnetic semiconductor. Science 301, 943–945 (2003). 12855816 10.1126/science.1086608

[b8] Chiba D. *et al.* Magnetization vector manipulation by electric fields. Nature 455, 515–518 (2008). 18818654 10.1038/nature07318

[b9] Daalderop G. H. O., Kelly P. J. & Schuurmans M. F. H. First-principles calculation of the magnetocrystalline anisotropy energy of iron, cobalt, and nickel. Phys. Rev. B 41, 11919–11937 (1990). 10.1103/physrevb.41.11919 9993644

[b10] Wang D.-s., Wu R. & Freeman A. J. First-principles theory of surface magnetocrystalline anisotropy and the diatomic-pair model. Phys. Rev. B 47, 14932–14947 (1993). 10.1103/physrevb.47.14932 10005868

[b11] Wu R. & Freeman A. Spin-orbit induced magnetic phenomena in bulk metals and their surfaces and interfaces. J. Magn. Magn. Mater. 200, 498–514 (1999).

[b12] Yosida K., Okiji A. & Chikazumi S. Magnetic anisotropy of localized state in metals. Prog. Theor. Phys. 33, 559–574 (1965).

[b13] Yosida K. Theory of magnetism. (Springer, Berlin; New York, 1996).

[b14] Meiklejohn W. H. & Bean C. P. New magnetic anisotropy. Phys. Rev. 105, 904–913 (1957).

[b15] Park M. S., Kwon S. K. & Min B. I. Electronic structures of doped anatase TiO_2_: Ti_1−*x*_*M_x_*O_2_ (*M* = Co, Mn, Fe, Ni). Phys. Rev. B 65, 161201 (2002).

[b16] Sullivan J. M. & Erwin S. C. Theory of dopants and defects in Co-doped TiO_2_ anatase. Phys. Rev. B 67, 144415 (2003).

[b17] Janisch R. & Spaldin N. A. Understanding ferromagnetism in Co-doped TiO_2_ anatase from first principles. Phys. Rev. B 73, 035201 (2006).

[b18] Persson C., Zhao Y.-J., Lany S. & Zunger A. *n*-type doping of CuInSe_2_ and CuGaSe_2_. Phys. Rev. B 72, 035211 (2005).

[b19] Raebiger H., Lany S. & Zunger A. Charge self-regulation upon changing the oxidation state of transition metals in insulators. Nature 453, 763–766 (2008). 18528391 10.1038/nature07009

[b20] Brooks H. Ferromagnetic anisotropy and the itinerant electron model. Phys. Rev. 58, 909–918 (1940).

[b21] Bruno P. Tight-binding approach to the orbital magnetic moment and magnetocrystalline anisotropy of transition-metal monolayers. Phys. Rev. B 39, 865–868 (1989). 10.1103/physrevb.39.865 9947253

[b22] Yang I., Savrasov S. Y. & Kotliar G. Importance of correlation effects on magnetic anisotropy in Fe and Ni. Phys. Rev. Lett. 87, 216405 (2001). 11736359 10.1103/PhysRevLett.87.216405

[b23] van der Laan G. Microscopic origin of magnetocrystalline anisotropy in transition metal thin films. J. Phys.: Condens. Matter. 10, 3239 (1998).

[b24] Matsumoto Y. *et al.* Room-temperature ferromagnetism in transparent transition metaldoped titanium dioxide. Science 291, 854–856 (2001). 11228146 10.1126/science.1056186

[b25] Chambers S. A. *et al.* Epitaxial growth and properties of ferromagnetic Co-doped TiO_2_ anatase. Appl. Phys. Lett. 79, 3467–3469 (2001).

[b26] Shinde S. R. *et al.* Ferromagnetism in laser deposited anatase Ti_1−*x*_Co*_x_*O_2−*δ*_ films. Phys. Rev. B 67, 115211 (2003).

[b27] Chambers S. A., Heald S. M. & Droubay T. Local Co structure in epitaxial Co*_x_*Ti_1−*x*_O_2−*x*_ anatase. Phys. Rev. B 67, 100401 (2003).

[b28] Li Y. *et al.* Gas sensing properties of p-type semiconducting Cr-doped TiO_2_ thin films. Sens. Actuators B 83, 160–163 (2002).

[b29] Ruiz A. M. *et al.* Cr-doped TiO_2_ gas sensor for exhaust NO_2_ monitoring. Sens. Actuators B 93, 509–518 (2003).

[b30] Kim C., Kim K.-S., Kim H. Y. & Han Y. S. Modification of a TiO_2_ photoanode by using Cr-doped TiO_2_ with an inuence on the photovoltaic efficiency of a dye-sensitized solar cell. J. Mater. Chem. 18, 5809–5814 (2008).

[b31] Dhoot A. S., Israel C., Moya X., Mathur N. D. & Friend R. H. Large electric field effect in electrolyte-gated manganites. Phys. Rev. Lett. 102, 136402 (2009). 19392377 10.1103/PhysRevLett.102.136402

[b32] Yoon S. D. *et al.* Nanogranular metallic Fe-oxygen deficient TiO_2−*δ*_ composite films: a room temperature, highly carrier polarized magnetic semiconductor. J. Phys.: Condens. Matter 20, 195206 (2008).

[b33] Perdew J. P., Burke K. & Ernzerhof M. Generalized gradient approximation made simple. Phys. Rev. Lett. 77, 3865–3868 (1996). 10062328 10.1103/PhysRevLett.77.3865

[b34] Kresse G. & Furthmüller J. Efficient iterative schemes for *ab initio* total-energy calculations using a plane-wave basis set. Phys. Rev. B 54, 11169–11186 (1996). 10.1103/physrevb.54.11169 9984901

[b35] Dudarev S. L., Botton G. A., Savrasov S. Y., Humphreys C. J. & Sutton A. P. Electron-energy-loss spectra and the structural stability of nickel oxide: An LSDA + U study. Phys. Rev. B 57, 1505–1509 (1998).

[b36] Walsh A., Da Silva J. L. F. & Wei S.-H. Theoretical description of carrier mediated magnetism in cobalt doped ZnO. Phys. Rev. Lett. 100, 256401 (2008). 18643681 10.1103/PhysRevLett.100.256401

