# Supplementary material for: Carrier-dependent magnetic anisotropy of cobalt doped titanium dioxide
Source: Sci Rep. 2014 Dec 16;4:7496. doi: 10.1038/srep07496 (PMC4266858; doi:10.1038/srep07496)
Supplement: Supplementary Information — Carrier-dependent magnetic anisotropy of cobalt doped titanium dioxide [file srep07496-s1.pdf]

Carrier-dependent magnetic anisotropy of cobalt doped titanium dioxide

Bin Shao[1], Min Feng[2], and Xu Zuo[3*]

[1]*State Key Laboratory of Low-Dimensional Quantum Physics, and Department of Physics Tsinghua University, Beijing 100084, China*

[2]*School of Physics, Nankai University, Tianjin 300071, China.*

[3]*College of Electronic Information and Optic Engineering, Nankai University, Tianjin 300071, China.*

[*] xzuonku@outlook.com

I. STABILITY OF FERROMAGNETISM AND EXCHANGE INTEGRALS

The stability of ferromagnetism was investigated by comparing the total energies of ferromagnetic (FM) and antiferromagnetic (AFM) states, for the neutral state ($\delta N = 0$) and a hole-accumulated state ($\delta N = -1.0$). To support the AFM calculations, the unit cell in the MAE calculation was doubled in the ab-plane. The resulting $\sqrt{2} \times \sqrt{2}$ and 1×2 unit cells are compatible with the checkerboard and row-by-row AFM, respectively (Fig. S1). The exchange integrals between the Co dopants is calculated by substituting the total energies of FM and AFM states into the Heisenberg model,

$$\mathcal{H} = -\frac{J_1}{2} \sum_{i,j \in nn} \mathbf{S}_i \cdot \mathbf{S}_j - \frac{J_2}{2} \sum_{i,j \in nnn} \mathbf{S}_i \cdot \mathbf{S}_j, \tag{S1}$$

where J_1 and J_2 are the exchange integrals between the nearest neighbors (nn) and the next nearest neighbors (nnn), respectively. In addition, local spin $S = 1/2$ and 1 are assumed for the neutral and charged states, respectively (Fig.1).

In the calculations of the FM stability and exchange integrals, the k-mesh is $6 \times 6 \times 6$ and the convergence criterion is 10^{-2} meV. In addition, extra on-site Coulomb repulsion ($U = 2$ eV) was applied on the d-orbitals of both Co and Ti ions, to enhance the localization underestimated by local-spin-density-based exchange-correlation.

The AFM energies are referenced to the FM and listed in Table I. The checkerboard AFM is the lowest and 2.13 meV lower than the FM, for the neutral state. However, the FM is the lowest and 43 meV lower than the checkboard AFM, for the charged state. This suggests that carrier accumulation can induce a transition of the magnetic ground state from AFM to FM, which is qualitatively agreed with the experimental observation [S1] and the theoretical prediction in Co:ZnO [S2]. The Curie temperature (T_C) of the charged state is estimated about 166 °K from the calculated J_1 by molecular field theory.

II. MAE AT FINITE TEMPERATURE

Following Ref. S3, we calculated MAE at finite temperature. In the calculation, we only considered the molecular field term and the D-term, and ignored higher order terms. We selected $S = 1$ that is approximately the spin per Co ion at $\delta N = -1.0$, where the magnetic anisotropy reaches the maximum. We did statistics and obtained the free energy of magnetic

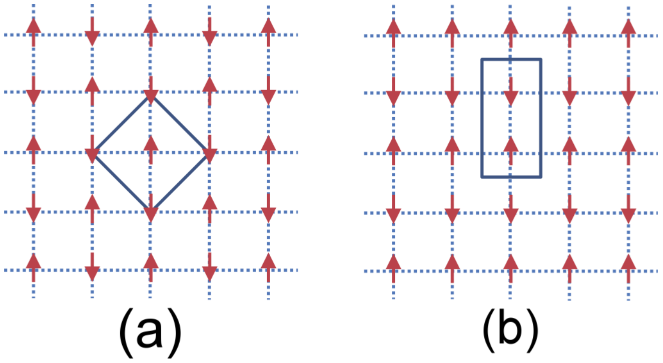

FIG. S1. Schematic illustration of the checkerboard (a) and row-by-row (b) antiferromagnetism in the ab-plane, where the $\sqrt{2}\times\sqrt{2}$ and 1×2 supercells indicated by the solid lines, respectively, and the lattice of Co ions is indicated by the dotted lines.

TABLE I. The AFM energies referenced to the FM and the exchange integrals in meV. For $\delta N = -1$, the calculation initiated with the row-by-row AFM rather converged to the non-magnetic state.

δ_N	checkerboard	row-by-row	J_1	J_2
0	-2.13	1.5	-1.07	1.28
-1	43	—	5.38	—

anisotropy as follows,

$$F_u = \frac{1}{6} N D (3\cos^2\theta - 1) p(y), \tag{S2}$$

where N is the number of ions, D is the coefficient of the D-term in the single-ion magnetic anisotropy Hamiltonian, θ is the angle between the spin-axis and anatase c-axis, and $p(y)$ is a rational function depending on molecular field and temperature as follows,

$$p(y) = \frac{1 - 2y + y^2}{1 + y + y^2}, \text{ and } y = \exp\left(-\frac{g\beta H_{\text{eff}}}{kT}\right), \tag{S3}$$

where H_{eff} is the molecular field. From equation S2, the magnetic anisotropy free energy is linearly proportional to the number of ions, N. It moreover depends on the molecular field and temperature via $p(y)$. We plotted $p(y)$ versus the relative magnetization, M/M_0, where M and M_0 are magnetizations at finite temperature and absolute zero, respectively. Fig. S2 shows that MAE will monotonically decrease as relative magnetization decreases and will

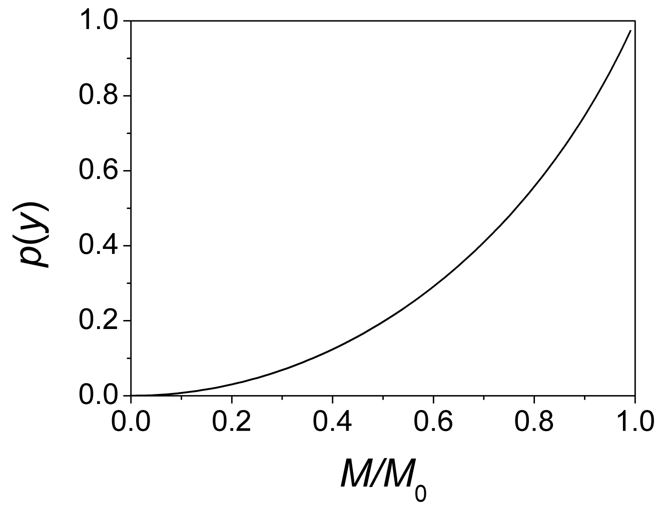

FIG. S2. $p(y)$ as a function of relative magnetization M/M_0.

vanish at $M/M_0 = 0$.

From the calculated MAE, we obtain the parameter $D = 3.2$ meV/Co in equation S2. Assuming the calculated $T_\mathrm{C} = 166\,^\circ$K, we predict that the MAE, via the $p(y)$ term in equation S2, will drop to 87% at the liquid nitrogen temperature (77 °K), and that the MAE of 5 Co dopants interacting each other in the square lattice will overcome the thermal fluctuation at this temperature. In addition, assuming the experimentally observed $T_\mathrm{C} \sim 400\,^\circ$K [S4], we predict that the MAE will drop to 47% at room temperature (300 °K), and that it will require 35 Co dopants, a 5 nm^2 area of the square lattice, to resist the thermal fluctuation.

[S1] Yamada, Y. *et al.* Electrically induced ferromagnetism at room temperature in cobalt-doped titanium dioxide. *Science* **332**, 1065–1067 (2011). URL `http://www.sciencemag.org/content/332/6033/1065.abstract`.

[S2] Walsh, A., Da Silva, J. L. F. & Wei, S.-H. Theoretical description of carrier mediated magnetism in cobalt doped zno. *Phys. Rev. Lett.* **100**, 256401 (2008). URL `http://link.aps.org/doi/10.1103/PhysRevLett.100.256401`.

[S3] Wolf, W. P. Effect of crystalline electric fields on ferromagnetic anisotropy. *Phys. Rev.* **108**, 1152–1157 (1957). URL `http://link.aps.org/doi/10.1103/PhysRev.108.1152`.

[S4] Matsumoto, Y. *et al.* Room-temperature ferromagnetism in transparent transition metal-doped titanium dioxide. *Science* **291**, 854–856 (2001). URL `http://www.sciencemag.org/content/291/5505/854.abstract`.
